# Fuelling prevention: federal levers to integrate nutrition into primary care

**DOI:** 10.1093/haschl/qxag027

**Published:** 2026-02-04

**Authors:** Robert L Phillips, Andrew W Bazemore, Garrett Kneese, Warren P Newton, Anand K Parekh

**Affiliations:** The Center for Professionalism & Value in Health Care, The American Board of Family Medicine, Inc., Washington, DC 20036, USA; The Center for Professionalism & Value in Health Care, The American Board of Family Medicine, Inc., Washington, DC 20036, USA; The Center for Professionalism & Value in Health Care, George Washington University, Washington, DC, USA; The American Board of Family Medicine, Inc. Lexington, KY 40511-1247, USA; University of Michigan, School of Public Health, Ann Arbor, MI, USA

**Keywords:** nutrition, primary care, prevention, medicare, health policy, family medicine, certifying board, medical education

## Abstract

Related to recent federal directives to strengthen physician nutrition education, this paper examines family medicine's leadership in nutrition counseling while identifying modifiable barriers limiting primary care's prevention potential. Family physicians, comprising over 109 000 certified clinicians, provide 20% of U.S. healthcare visits and deliver substantial nutrition counseling, particularly in underserved communities. The American Board of Family Medicine dedicates 5% of certification content to nutrition/obesity and 25% to chronic disease care. However, structural barriers significantly constrain implementation. Medicare only reimburses nutrition counseling for end-stage conditions (diabetes and kidney disease), frustrating key opportunities to help patients. Primary care receives <5% of national health spending and under 1% of federal research funding, despite handling half of all office visits. The workforce lacks integrated nutritionists and dietitians, with physicians 20 times more likely to address nutrition when services are covered. We propose federal actions to transform primary care's nutrition capacity: expanding Medicare coverage to earlier disease stages and obesity, increasing primary care investment, directing research investment to community practice settings, supporting workforce integration of nutrition professionals, and developing meaningful quality measures. These aligned policies could unleash primary care's prevention potential.

## Introduction

The Secretary of Health and Human Services recently called on physician education, certification organizations to strengthen the knowledge, and skills of physicians in nutrition and chronic condition prevention.^1^ These organizations were given 2 weeks to submit “written plans detailing the scope, timeline, standards alignment, measurable milestones, and accountability measures of their nutrition education commitments.” It is worth noting that this was already a bipartisan Congressional issue and a focus of the Bipartisan Policy Center.^2,3^ We highlight progress and persistent barriers that constrain primary care's role in nutrition counseling, and propose federal actions that could accelerate transformation.

We highlight Family Medicine because it is the most broadly distributed medical discipline among those charged with this task. Family Physicians provide more than 20% of all U.S. health care visits, are the largest physician workforce in underserved and rural communities, and deliver much of the nutrition counseling that occurs in primary care.^4^ With over 109 000 family physicians currently certified by the American Board of Family Medicine (ABFM), certification standards directly influence training requirements and clinical practice competencies. A self-regulatory body with responsibility to ensure a higher standard to the public and patients, the ABFM has a mission to align certification with public health priorities and the highest standards of guideline-based care. Through the Center for Professionalism and Value in Health Care, ABFM also seeks to strengthen the social contract between medicine and society and improve clinician capacity to deliver high-value care. Improving nutrition counseling and chronic condition prevention is certainly pertinent to physician certification, but the constraints on delivering this care are not usually in the physician's control to change. Federal action could be enabling.

## Gaps in nutrition education

Despite recognition of its importance, nutrition education across medical training remains inconsistent and insufficient. Most U.S. medical students receive fewer than 20 hours of nutrition instruction over 4 years and these gaps extend into residency and practice.^5^

Family medicine, however, has been at the forefront of efforts to address this shortcoming. Programs such as the University of North Carolina's Nutrition in Medicine curriculum demonstrate scalable models that can elevate nutrition as an educational priority.^6^ Professional organization continuing education resources, like the American Academy of Family Physicians evidence-based module, *Diets for Health: Goals and Guidelines*, support lifestyle counseling in routine care.^7^ The ABFM certification blueprint devotes ∼5% of its examination content to nutrition and obesity, and more than 25% to chronic disease care. Additional certification requirements include clinical activities focused on nutrition/disease prevention, and nearly a quarter of sports medicine practice activities incorporate exercise, diet, or prevention. Together, these standards help ensure that family physicians encounter nutrition content throughout training, examination, and ongoing certification, signaling its importance as a professional competency.

## Structural barriers that limit progress

Despite these efforts, several systemic barriers prevent primary care from fully realizing its potential in nutrition and prevention. These include the following:.

### Coverage

Medicare currently only reimburses nutrition counseling for patients with diabetes, kidney disease, or kidney transplants—essentially end-stage disease. Expanding coverage to earlier disease stages and obesity could prevent progression and reduce costs. Evidence shows that family physicians were 20 times more likely to refer patients for nutrition support when such services were covered, but referrals reverted to baseline when coverage was withdrawn.^8^ While insurance coverage of obesity management and treatment by state employee plans has improved over the last decade, few of these patients receive appropriate lifestyle and nutrition counseling or treatment suggesting that real barriers remain in place. The proposed Medical Nutrition Therapy Act could be a real boon to the Secretary's goals through expanded coverage.

A recent CMS Innovation Center Notice of Funding Opportunity aims to test care models with enhanced capacity to address nutrition, fitness, sleep, and stress management. Making America Healthy Again: Enhancing Lifestyle and Evaluating Value-based Approaches Through Evidence offers moderate funding to 30 sites for 3 years to help Medicare beneficiaries with these additional services.^9^ The Massachusetts Delivery System Reform Incentive Payment program created the Flexible Services Program, which pays for health-related nutrition education and supplementation.^10^ A recent NASEM Primary Care Standing Committee response to CMS suggested that the Advanced Primary Care Management (APCM) hybrid payment model be enhanced to provide funding needed to deliver or support nutrition counseling.^11^ The APCM is already designed to improve chronic care management. These examples of specific funding options could lead to better coverage of patient nutrition support and be replicated by other payers.

### Investment

Primary care is chronically underfunded. The 2021 National Academies of Sciences, Engineering, and Medicine primary care report urged increasing primary care's share of national health spending from under 5% to at least 10% to sustain comprehensive, team-based care, including embedded nutrition services. The report offers a model of the team and functions that should be part of comprehensive primary care. This included community-based functions focused on a patient's whole health. This model was embraced by 2 different CMS Innovation Center payment models but without adequate work to price what it costs to deliver the expected services. While achieving this is a goal of doubling primary care spending, it is possible to do more objective pricing of the model and services for sufficient coverage. Whether through simple doubling or objective pricing, without increased investment, primary care will remain stretched too thin to integrate preventive counseling at scale. While it is possible to increase education or impose nutrition counseling quality measurement, both are likely to have weak effect without supporting investment for these services. Likewise, training more nutritionists or care managers will not induce them to work in primary care or partnering community-based organizations without sufficient payment to make them viable careers. It is important to acknowledge Medicare budget neutrality requirements, but it is these very cost offsets that have reduced investment in primary care over time. It is time to rebalance health care investments and focus on the sector that has proven capacity to reduce downstream costs through prevention and chronic care management.

### Research

Primary care visits comprise half of all office visits (and Family Medicine about half of these), but <1% of federal research funding flows to primary care and only 0.2% supports research in family medicine. While primary care researchers study nutrition and obesity interventions in real-world settings, federal priorities overwhelmingly favored subspecialty and hospital-based research. Without dedicated funding in, for, and by primary care, evidence for nutrition intervention implementation in community practice will remain weak. HHS could repair this mismatch of research support for where most people receive care and where chronic conditions are addressed before they are more costly. Recently, the National Institutes of Health has appeared willing to adjust its research funding priorities and alignment with the Secretary's goals for whole health approaches in primary care would be supportive.

### Workforce

Federal support for integrating registered dietitians, nutritionists, and community health workers into primary care could extend counseling capacity and multiply the effectiveness of primary care. The evidence for impact of nutritionists and dieticians in primary care is compelling but Medicare and other payers do not support this workforce integration. Further to the Secretary's goals, loan repayment programs or targeted funding streams could encourage placement of these professionals in underserved areas and in integrated practices. For example, the Health Resources and Services Administration have limited awards for disadvantaged students studying to become registered dieticians, offering a vehicle for workforce if expanded.

### Measurement

Nutrition counseling and outcomes are inconsistently tracked in quality reporting systems. CMS and other agencies should develop measures that capture counseling frequency, patient weight loss, or population-level nutrition indicators. Inclusion in value-based payment models would create accountability and reward prevention. However, these measures should not add to physician burden without increasing support for these services embedded in practices. We summarize barriers and the policy options to address each in Table 1.

**Table 1.** Policy opportunities for strengthening nutrition and prevention in primary care.

Policy lever	Current gap	Federal action needed	Expected impact
Insurance Coverage for Nutrition counseling in Primary Care	Nutrition counseling only covered for diabetes and kidney disease under Medicare	Expand coverage to counseling for all patients, not just those with end-stage disease	Earlier intervention, reduced progression to chronic disease, cost savings
Increase Primary Care Investment	<5% of national health spending goes to primary care	Implement NASEM recommendation to increase to ≥10% or price the comprehensive care model needed in primary care; reverse cost offsets that have starved primary care	Enable team-based care capacity, integrate nutrition counseling at scale
Increase Research Funding for Primary Care	<1% of federal research dollars directed to primary care	Prioritize research in primary care with emphasis on lifestyle and Nutritional counseling, and Implementation Science	Build evidence base for effective nutrition interventions in real-world settings
Nutrition Workforce Integration into PC teams	Lack of embedded nutrition professionals in primary care	Fund dietitian/community health worker integration via loan repayment or grants; enable coverage to fund these roles and make them viable careers	Extend counseling reach, multiply physician capacity
Nutrition Quality Measurement	Inconsistent tracking of nutrition services and outcomes	Develop and adopt primary care nutrition counseling metrics in CMS and value-based payment programs but only if aligned with sufficient resources to deliver desired care	Incentivize prevention, support accountability

## Conclusion

Family physicians are well trained to deliver nutrition and prevention, all reinforced in their certification standards, and stand ready to deliver on national prevention goals. Expanding coverage, increasing primary care investment, boosting research support, strengthening the workforce, and measuring what matters would together transform primary care's capacity to address nutrition and prevention. Family medicine is prepared to lead but systemic barriers must be reduced to unleash its full potential.

## Supplementary Material

qxag027_Supplementary_Data
